# Correlates of Engagement Within an Online HIV Prevention Intervention for Single Young Men Who Have Sex With Men: Randomized Controlled Trial

**DOI:** 10.2196/33867

**Published:** 2022-06-27

**Authors:** Seul Ki Choi, Jesse Golinkoff, Mark Michna, Daniel Connochie, José Bauermeister

**Affiliations:** 1 Department of Family and Community Health School of Nursing University of Pennsylvania Philadelphia, PA United States

**Keywords:** paradata, mobile health, mHealth, digital health intervention, risk reduction, HIV prevention, public health, digital health, sexual health, sexual risks

## Abstract

**Background:**

Digital HIV interventions (DHI) have been efficacious in reducing sexual risk behaviors among sexual minority populations, yet challenges in promoting and sustaining users’ engagement in DHI persist. Understanding the correlates of DHI engagement and their impact on HIV-related outcomes remains a priority. This study used data from a DHI (myDEx) designed to promote HIV prevention behaviors among single young men who have sex with men (YMSM; ages 18-24 years) seeking partners online.

**Objective:**

The goal of this study is to conduct a secondary analysis of the myDex project data to examine whether YMSM’s online behaviors (eg, online partner-seeking behaviors and motivations) are linked to participants’ engagement (ie, the number of log-ins and the number of sessions viewed).

**Methods:**

We recruited 180 YMSM who were randomized into either myDEx arm or attention-control arm using a stratified 2:1 block randomization. In the myDEx arm, we had 120 YMSM who had access to the 6-session intervention content over a 3-month period. We used Poisson regressions to assess the association between YMSM’s baseline characteristics on their DHI engagement. We then examined the association between the participants’ engagement and their self-reported changes in HIV-related outcomes at the 3-month follow-up.

**Results:**

The mean number of log-ins was 5.44 (range 2-14), and the number of sessions viewed was 6.93 (range 0-22) across the 3-month trial period. In multivariable models, the number of log-ins was positively associated with high education attainment (estimated Poisson regression coefficient [β]=.22; *P*=.045). The number of sessions viewed was associated with several baseline characteristics, including the greater number of sessions viewed among non-Hispanic YMSM (β=.27; *P*=.002), higher education attainment (β=.22; *P*=.003), higher perceived usefulness of online dating for hookups (β=.13; *P*=.002) and perceived loneliness (β=.06; *P*=.004), as well as lower experienced online discrimination (β=–.01; *P*=.007) and limerence (β=–.02; *P*=.004). The number of sessions viewed was negatively associated with changes in internalized homophobia (β=–.06; *P*<.001) and with changes in perceived usefulness of online dating for hookups (β=–.20; *P*<.001). There were no significant associations between the number of log-ins and changes in the participants’ behaviors at the 90-day follow-up.

**Conclusions:**

DHI engagement is linked to participants’ sociodemographic and online behaviors. Given the importance of intervention engagement in the intervention’s effectiveness, DHIs with personalized intervention components that consider the individuals’ differences could increase the overall engagement and efficacy of DHIs.

**Trial Registration:**

ClinicalTrials.gov NCT02842060; https://clinicaltrials.gov/ct2/show/NCT02842060.

## Introduction

HIV infections among young men who have sex with men (YMSM) between 13 and 29 years of age are of particular concern in the United States [1]. HIV prevention digital health interventions (DHI) provide opportunities to reach YMSM and offer HIV-related prevention information given their appeal and broad reach [2]. High technology use among youth makes DHIs feasible, enables easier and faster spread of information, offers a greater number of opportunities for real-time behavior change cues and nudges, and provides greater access to social support and engagement, particularly for individuals who might experience stigma in their real-world environments [3-5]. By design, DHIs are appealing because they can be delivered remotely, allow for self-guided learning, and encourage asynchronous interaction with others. As a result, evaluating the effectiveness of DHIs requires a different set of considerations, as compared to face-to-face interventions that are delivered by a facilitator in a specific time and place. Researchers have recently noted how these engagement considerations remain the crucial factor in evaluating the true intervention effects of DHIs [5].

DHIs have been linked to changes in cognitive and behavioral risk factors, increases in the adoption of HIV prevention behaviors, and the development of supportive relationships online [6,7]. While the strengths of DHIs are noteworthy, a recent review [8] of 16 DHI studies on HIV prevention and treatment (8 studies encouraged HIV testing, 7 studies targeted condom use, 3 studies promoted preexposure prophylaxis initiation and adherence, and 3 studies encouraged antiretroviral therapy adherence) among gay, bisexual, and other men who have sex with men (MSM) published between 2012 and 2019 found that 33% of the interventions that intended to promote HIV testing and 43% of those that intended to increase condom use were not statistically effective [8]. The absence of observed effects in these interventions may be related to participants’ engagement with the interventions. In a recent review, Hightow-Weidman and Bauermeister [9] documented how participants’ engagement with DHI content was associated with key HIV prevention outcomes across 4 distinct HIV interventions designed for YMSM. They found that intervention exposure and dosage, between-arm and within-arm, strengthened the observed intervention effects.

Limited engagement can impact an intervention’s effect on behavior change; however, it is imperative that researchers examine participants’ engagement with DHI to enhance the precision in calculating the efficacy of their interventions and ultimately maximize the effectiveness and efficiency of their interventions. For example, researchers found that engagement moderated the efficacy of healthMpowerment.org (HMP), a theory-based phone-optimized DHI for young Black MSM. Participants who met the recommended engagement time with the intervention (ie, 60 minutes or more during the 3-month intervention period) showed greater reduction in the number of condomless anal intercourse (CAI) episodes compared to those who did not comply with the recommended engagement time [7]. Moreover, the total time spent on HMP was correlated with overall site satisfaction during usability assessment [10], and participants who engaged with the intervention components where those who could share experiences and receive social support (eg, Forum, Getting Real, and Ask Dr.W), and the content of the intervention exhibited reduced levels of stigma [11]. Therefore, without engagement metrics, it is hard to know whether a DHI was delivered to participants, achieving the intervention “dose” required for optimal behavior change, as these applications offer an array of different activities and features without dictating a standardized sequence of activities, amount of exposure or frequency, and duration of interactivity.

Researchers have promoted the use of *paradata* metrics for measuring engagement with DHIs [9,12]. *Paradata* can be defined as automatically generated process data that capture participants’ actions within an application [13-15], and can be transformed to characterize the amount, frequency, duration, and depth of engagement across and within DHIs [9]. Thus, *paradata* metrics are crucial to understanding how differential engagement might impact behavior change and help inform what constitutes meaningful engagement [16]. To date, limited attention has been paid to whether participants’ characteristics may serve as correlates of DHI engagement. Several recent studies have noted that participants’ sociodemographic characteristics may be associated with DHI engagement [17,18]. Beyond examining sociodemographic differences in DHI engagement, few studies have examined whether other psychosocial factors are related to DHI engagement. Understanding the antecedents to DHI engagement may help researchers and practitioners alike to create implementation strategies that improve engagement and, in turn, maximize its potential effects.

In order to characterize users’ engagement, this study examines how the interplay of internet use patterns and partner-seeking characteristics influence engagement in DHIs. Therefore, the goal of this study is to conduct a secondary analysis of the myDex project data to examine whether YMSM’s online behaviors (eg, online partner-seeking behaviors and motivations) are linked to participants’ engagement with the DHI. To advance this goal, our study had 3 objectives. First, we examined whether YMSM’s internet-using patterns, relationship characteristics, psychological facilitators and barriers, and sexual behaviors predicts their DHI engagement. Second, we explored whether participants’ engagement during the 90-day intervention impacted psychobehavioral changes in internet use patterns, relationship characteristics, psychological facilitators and barriers, and sexual behaviors from baseline to the 90-day follow-up. Third, we evaluated whether there are different correlates between frequency of engagement (number of log-ins) and amount of engagement (number of sessions viewed).

## Methods

### Ethics Approval

The research and ethics presented in this study have been reviewed and approved by the University of Michigan Institutional Review Board (HUM00091627). The University of Pennsylvania ceded regulatory oversight to the University of Michigan (University of Pennsylvania IRB #824885). The study is also registered on ClinicalTrials.gov (NCT02842060).

### Study Procedure

**Figure 1:**
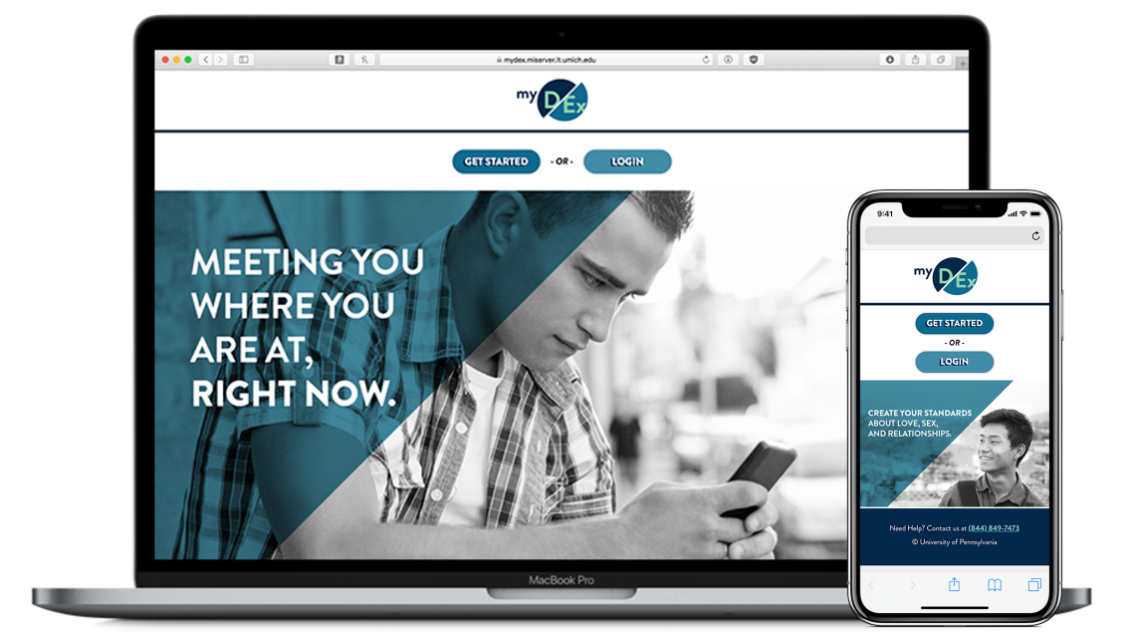
Screenshot of myDEx intervention.

Data from this study come for the myDEx web application, a DHI trial delivering dating and partner-seeking behavior content for single YMSM presumed to be HIV-negative and who engage in CAI with sexual partners met online (Figure 1). A detailed protocol for myDEx has been outlined elsewhere [19]. The participants were recruited across the United States through advertisements on online social media and sexual networking platforms. Social network advertisements were targeted to men who fit the study’s age criterion and who lived in the United States.

To participate, participants had to self-report the following: (1) male sex at birth and male gender identity; (2) age of 18 to 24 years; (3) HIV-negative or HIV-unaware serostatus; (4) single relationship status; (5) prior use of online dating applications; and (6) report CAI with at least one male partner in the prior 6 months. Upon completion of an online informed consent form, eligible participants completed a 30-minute web-based baseline questionnaire ascertaining their sexual and online behaviors, mental health, and demographic information.

A sample of 180 single YMSM (aged 18-24 years; 50% [n=90] racial or ethnic minorities) were recruited between November 2016 and January 2017 and randomized to either the intervention arm (myDEx) or the attention-control arm using a stratified 2:1 block randomization design.

The participants were given access to myDEx for 90 days. The intervention (myDEx) was divided into 6 sessions, each addressing distinct cognitive and affective content areas (Table 1). Within each session, intervention content was organized into the following three levels: (1) core messages, (2) in-depth discussion of topics linked to the core message, and (3) an interactive activity linked to the information presented. Within each session, the participants had access to brief activities and videos designed to build their HIV risk reduction skills and promote self-reflection about their sexual health and partner-seeking behaviors. We designed the sessions to keep users engaged for at least 10 minutes. The participants were required to complete the first session before being able to access the other 5 sessions and interactive activities [19]. The participants could view the sessions multiple times. However, we did not have a priori threshold for the number of sessions viewed and log-ins, nor did we set an expectation for users to use the intervention over a number of sessions or log-ins. This study analyzed (1) the effect of baseline characteristics on engagement in the myDEx intervention over 90 days and (2) the associations between engagement in the myDEx intervention as well as changes in the participants’ characteristics during 90 days among 120 single YMSM in the myDEx intervention arm.

**Table 1:** Content of 6 sessions in myDEx.

Session	Content
Session 1: “Sexuality & Relationships”	The importance of feeling comfortable talking about sexuality, desires within relationships, and health
Session 2: “Desires & Behaviors”	Different relationship types (eg, romantic relationships, friends with benefits, and hookups) and sexual decision-making
Session 3: “What Makes Good Sex”	Comprehensive sex education: same-sex behaviors, including the importance of sex positivity, varying sexual practices, and sexual consent
Session 4: “Sexual Well-being”	HIV and STI^a^ risks reduction when engaging in anal sex: (1) what lubricants and condoms are best suited for anal intercourse; (2) facts about HIV and STI transmission; and (3) the importance of status disclosure prior to sex.
Session 5: “Getting The Sex You Want”	Strategies to improve sexual communication with partners before, during, and after sex
Session 6: “Your Body, Your Health”	Summarizes key messages from prior modules; offers nearby HIV/STI testing resources and PrEP^b^ locations.

^a^STI: sexually transmitted infection.

^b^PrEP: preexposure prophylaxis.

### Measures

This study analyzed the myDEx intervention arm (n=120) *paradata* over 90 days, participant characteristics at baseline, and participant characteristics at the 90-day follow-up. Participants characteristics were examined for associations with intervention engagement.

#### Participant Paradata

Over the 90-day trial period, the participants’ actions in myDex were collected as *paradata*. *Paradata* can be transformed to characterize the amount, frequency, duration, and depth of engagement with a web-based intervention [9]. Amount refers to a quantity of something in number, size, or value. Frequency is the number of occurrences of a repeating event over a particular time. Duration is the time during which something continues. Depth represents the usage of different intervention components. In this study, we employed two types of *paradata* metrics, which are (1) the frequency of engagement (number of log-ins) and (2) the amount of engagement (number of sessions viewed). We measured the frequency of intervention use by counting the number of log-ins during the intervention period and the amount by counting the number of sessions viewed per log-in.

#### Demographic Characteristics

We asked the participants to report their age and ethnicity. In addition, the participants were asked to report their highest level of education (some high school, graduated high school, technical school, associate degree, some college, college, some graduate school, or graduate degree). Then, education was dichotomized as “less than associate degree” or “associate degree, college graduate, or more than college.”

#### Internet Use Patterns

##### Frequency and Usefulness of Online Dating

The participants were asked about frequency and usefulness of online dating to find a date, and the same set of questions were asked regarding finding a hookup in the past 30 days. The frequency of engaging in online dating had the following six response options: (1) “Never,” (2) “Once a month or less,” (3) “2-3 times a month,” (4) “About once a week,” (5) “2-6 times a week,” and (6) “About once a day.” The usefulness of using online dating employed a 4-point Likert-type scale from “Not at all” to “Very much.”

##### Online Discrimination

We used an 8-item adapted version of the Everyday Discrimination Scale [20] to measure experienced discrimination when looking for partners online (α=.81). The example items were as follows: “People act as if they think you are not smart” and “You are treated with less courtesy than other people are.” The response had 6 response options ranging from “Never” to “Almost every day.” We created a continuous score by summing 8 items (range 0-40), with higher scores indicating higher experienced discriminations when looking for a partner online.

#### Psychological Facilitators and Barriers

##### Internalized Homophobia

We used a 7-item, revised Reactions to Homosexuality Scale [21] to measure internalized homophobia. The scale includes statements such as “Even if I could change my sexual orientation, I wouldn’t” and “I feel comfortable being a homosexual man.” Scoring is reversed for 1 item, which is positive affect statements. The scale employed a 5-point Likert scale with response options from Strongly Disagree to Strongly Agree. Then, total score was computed by creating sum score (range 5-35) with higher scores indicating higher internalized homophobia (α=.72).

##### Loneliness

We used the 3-Item UCLA Loneliness Scale to measure overall social isolation [22]. The items were as follows: (1) “How often do you feel that you lack companionship?” (2) “How often do you feel left out?” and (3) “How often do you feel isolated from others?” The response categories were coded 1=hardly ever, 2=some of the time, and 3=often. We used the sum scores of these 3 items, with higher scores indicating greater social isolation (range 3-9; α=.84).

##### Mental Health

We used the Center for Epidemiologic Studies Depression Scale with 10 items to measure mental health status in the past week [23]. The scale includes 3 items on depressed affect, 5 items on somatic symptoms, and 2 on positive affect. The scale employed a 4-point Likert scale ranging from “rarely or none of the time” to “all of the time.” Scoring is reversed for 2 items (“I felt hopeful about the future” and “I was happy”), which are positive affect statements. Total scores can range from 0 to 30 (α=.83), with higher scores indicating greater severity of symptoms.

##### Self-esteem

Rosenberg et al [24] developed a scale with 10 items (eg, “On the whole, I am satisfied with myself”) with responses rated on a 4-point Likert-type scale (“strongly agree” to “strongly disagree”) to estimate individuals’ self-esteem. Scoring was reversed for negatively worded items. A higher score indicates greater self-esteem (range 0-30; α=.90).

#### Relationship Characteristics

##### Ideal Relationship Characteristics (Intimacy, Commitment, and Passion)

We used the Triadic Love Scale to assess YMSM’s perceived relationship characteristics [25]. The participants responded to the importance of quality in their ideal romantic relationship with their partner. The original scale with 20-item employs a 4-point Likert-type scale with response options ranging from “Not at all important” to “Very important.” Three subscales were derived from the following scales: intimacy (eg, “To feel close to your partner”; 9 items; α=.90); commitment (eg, “To feel a sense of responsibility towards your relationship”; 5 items; α=.75); and passion (eg, “To explore your sexuality with your partner”; 6 items; α=.82). In this study, we computed a mean score for each subscale (range 1-4), where higher scores indicate greater ideation on that component.

##### Limerence

We adapted a limerence scale to measure the intense feelings of dependence, insecurity, and doubt about a relationship and experiences with intrusive and intense thoughts about partners [26]. We asked the participants 8 items using a 5-point scale ranging from 1 (“Strongly disagree”) to 5 (“Strongly agree”). The scale includes statements such as “I think about how being in a relationship would solve my problems,” “I have sex to feel loved,” and “I obsess about a specific person even though it may not work out.” We computed a score summing 8 items ranging from 8 to 40, where higher scores indicate greater limerence (α=.84).

#### Sexual Risk Behaviors

##### Decisional Balance to Condom Use

We used the Decisional Balance Scale to examine the participants’ decisional balance to use or forego condoms with partners [27]. The participants were asked 7 paired statements. For each item, the participants rated their preference for sex without condoms, followed by the same question asking about preference for sex with condoms. The items included “Sex [with/without] condoms is very intimate to me” and “Sex [with/without] condoms makes me feel close to my partner.” Each item was measured using a 4-point scale ranging from “Strongly disagree” to “Strongly agree.” A net difference for decisional balance items was created by summing the net difference between condomless sex and condom use scores across the items, resulting in 7 net scores ranging from –3 to +3. Finally, we created the total decisional balance to use condoms scores by computing a mean score of these 7 items. Positive scores indicate greater endorsement of sex without condoms, while scores close to zero indicate a decisional balance between sex with and without condoms (α=.89).

##### Self-efficacy to Use Condoms

We used an 8-item scale to measure how hard or easy it is to use condoms with a date (α=.82), respectively, and the same set of questions was used for a hookup (α=.77). The example items were as follows: “To have condoms with you in case you have sex?” and “To discuss having safer sex with a hookup partner online?” The self-efficacy to use condoms scale employed a 4-point Likert-type scale from “Very easy to do” to “Very hard to do” (range 8-32). The total self-efficacy to use condoms was computed by summing the scores of these 8 items. Higher scores indicate hardship in using condoms when thinking about a date or a hookup.

##### The Number of Sex Partners and Anal Intercourse

We used an adapted version of the Sexual Practices Assessment Schedule [28,29] to quantify the number of male partners in the prior 30 days. First, the participants indicated the total number of male sexual partners with whom they had sex (oral or anal). Then, they were asked to report the number of male sexual partners with whom they had receptive and insertive anal sex. Lastly, the participants were asked to indicate the number of partners with whom they did not use condoms. We created a continuous variable to measure the number of sex partners and the number of engagements in receptive or insertive anal intercourse. We excluded outliers for the number of sex partners and anal intercourse.

### Statistical Analysis

Descriptive statistics were used to summarize the study participants’ characteristics including internet use patterns, relationship characteristics, psychological facilitators and barriers, and sexual behaviors. Differences in the participants’ characteristics between baseline and 90-day follow-up were compared using McNemar tests and paired *t* tests. Then, we used Poisson regressions with robust variance to assess the effect of the participants’ baseline characteristics on 2 engagement outcomes (ie, the number of sessions viewed and the number of log-ins) and the associations between changes in the participants’ characteristics and engagement within the myDEx intervention for 90 days. Multivariable models were fitted based on significant variables in bivariable models (*P*<.05). All analyses were conducted in SAS 9.4 (SAS Institute Inc) [30].

## Results

### Description of the Study Participants

We summarized the participants’ characteristics in Multimedia Appendix 1. Among 120 participants, the mean age was 21.67 (SD 1.81) years. Most participants were identified as White (n=89, 74.2%), followed by Black (n=18, 15.0%), Other (n=12, 10.0%), and Asian (n=10, 8.3%). One-third of the participants (n=35, 29%) were Latino, and most participants (n=98, 81.67%) received education of some associate degree or higher. A majority of participants (n=91, 75.8%) used the internet for 1 to 6 hours per day. Almost half of the participants used online dating at least once a week to find a date (n=59, 49.2%), while a majority of participants used online dating less than 2-3 times a month to find a hookup (n=100, 83.2%). However, the participants considered online dating a useful tool to find a hookup (n=55, 45.8%) rather than a date (n=36, 30%). They also experienced moderate levels of discrimination in online settings. In addition, they showed a propensity toward seeking out novel or risky sexual stimulation (mean 20.5, SD 7.8) and had moderate ideation on intimate (mean 3.8, SD 0.3), passionate (mean 3.6, SD 0.4) and committed (mean 3.7, SD 0.4) relationships. Additionally, they reported the intense feelings of dependence, insecurity, and doubt about a relationship as well as experiences with intrusive and intense thoughts about partners (mean 22.9, SD 6.6).

We summarized engagement in the myDEx intervention over 90 days with the frequency of engagement (number of log-ins) and the amount of engagement (number of sessions viewed). On average, the participants logged into the myDEx intervention 5.44 times (range 2-14) and viewed sessions 6.93 times (range 0-22) during the 90 days of intervention.

### Baseline Characteristics and myDEx Engagement

#### The Number of Log-ins

In bivariable models (Multimedia Appendix 2), the participants were more likely to log into myDEx during the 90 days of intervention if they had higher educational attainment (estimated Poisson regression coefficients [β]=.23; *P*=.04) and reported higher frequency of online dating to find a hookup (β=.07; *P*=.03), higher perceived usefulness of online dating for a hookup (β=.09; *P*=.01), greater loneliness (β=.05; *P*=.02), and higher number of sex partners (β=.04; *P*=.003).

In a multivariable model, higher education attainment (β=.22; *P*=.045) and loneliness (β=.04; *P*=.07) remained associated with the number of log-ins during the intervention.

#### The Number of Sessions Viewed

Similar to the number of log-in models, in bivariable models, the participants who identified as Hispanic (β=–.25; *P*=.002) and reported higher discrimination experiences in an online setting (β=–.01; *P*=.02) and limerence (β=–.01; *P*=.02) at baseline viewed fewer sessions. However, the participants viewed more sessions after the 90-day intervention if they had higher educational attainment (β=.25; *P*=.002), reported higher frequency of online dating use to find a hookup (β=.06; *P*=.02), perceived greater usefulness of online dating to find a hookup (β=.14; *P*<.001), experienced greater loneliness (β=.05; *P*=.01), and had a greater number of sex partners (β=.04; *P*=.001) at baseline.

In a multivariable model, the number of sessions viewed was associated with non-Hispanic ethnicity (β=–.27; *P*=.002), higher educational attainment (β=.22; *P*=.003), perceived usefulness of online dating for hookups (β=.13; *P*=.002), loneliness (β=.06; *P*=.004), experienced online discrimination (β=–.01; *P*=.007), and limerence (β=–.02; *P*=.004).

### Changes in the Participants’ Behaviors Based on myDEx Engagement

At the 90-day follow-up, the participants’ frequency of online dating to find a date or a hookup decreased significantly (Multimedia Appendix 1). At baseline, 12.5% (n=15) of the participants had not used online dating to find a date in the past month, but at the 90-day follow-up, 33.7% (n=32) of the participants had not used online dating to find a date in the past month (*P*=.004). Similarly, 20% (n=24) of the participants never used the internet to find a hookup at baseline, but this percentage increased to 46.3% (n=44) at the 90-day follow-up (*P*=.007). In addition, their experienced discrimination in an online setting decreased significantly from baseline to the 90-day follow-up (baseline mean 17.0; and 90-day follow-up mean 3.25; *P*<.001). The participants also showed improvements in their decisional balance of having sex with and without condoms (baseline mean –0.42; and 90-day follow-up mean –0.26; *P*=.03) and reported fewer sex partners in the past month (baseline mean 2.39; and 90-day follow-up mean 1.15; *P*<.001). We examined whether these changes over time were correlated with YMSM’s engagement with the DHI.

#### The Number of Log-ins

There were no significant associations in bivariate or multivariable models between the number of log-ins and changes in the participants’ behaviors at the 90-day follow-up (Multimedia Appendix 3).

#### The Number of Sessions Viewed

In bivariate models, the number of sessions viewed was negatively associated with the perceived usefulness of online dating for hookups (β=–.21; *P*<.001) and internalized homophobia (β=–.03; *P*=.008). However, the number of sessions viewed were positively associated with increased ideation of an intimate romantic relationship (β=–.29; *P*=.04) and increased number of insertive anal intercourse events (β=.08; *P*=.02).

In the multivariable model, the number of sessions viewed was negatively associated with internalized homophobia (β=–.06; *P*<.001) and with changes in perceived usefulness of online dating for hookups (β=–.20; *P*<.001). No other statistically significant associations were observed.

## Discussion

### Principal Results

DHIs have great potential for HIV prevention, but there is divergence in their effectiveness in the existing literature [8]. The discrepancy in DHI effectiveness may be attributable to variations in the participants’ engagement. Therefore, researchers have recently noted how engagement considerations are a crucial factor in evaluating the true intervention effects of DHIs [12,31,32]. In this study, we elucidated whether DHI engagement as defined by 2 *paradata* indicators (ie, frequency of log-ins and number of sessions viewed) are associated with participants’ characteristics and the intervention’s effect on several HIV-related behavior at the 90-day follow-up.

The participants who engaged in the myDEx intervention logged in at least 2 times, with a maximum of 14 times, in the 90-day intervention period. Moreover, the participants viewed an average of 7 sessions. However, there were 8/120 (6.7%) participants who never viewed any of the sessions, including the initial mandatory session. Varied engagement was driven by differences in the participants’ sociodemographic characteristics and online behaviors. Similar to the study by Bonett et al [17], we found that both frequency and amount of engagement were greater among YMSM with higher educational attainment. We also noted lower amounts of engagement among Latino participants. DHIs have the potential to reduce HIV inequities among underserved communities, including racial and ethnic minority communities and populations with fewer socioeconomic resources [33]; however, our findings suggest that these inequities may not be resolved if the same populations are less likely to engage with DHIs. Efforts to address the digital divide by addressing health literacy [34], cultural competency [35], and high-quality access to technologies that facilitate DHI engagement are warranted. We recommend that future intervention studies examine the extent to which increasing health literacy and cultural factors as well as addressing online access barriers (eg, reducing entry barriers) may be warranted [36-38] to increase engagement among underserved populations that could benefit from DHIs.

Engagement was also linked to YMSM’s online partner-seeking behaviors at baseline. Engagement was greater among YMSM who perceived online dating applications as a useful hookup tool and who self-reported interpersonal difficulties both online and offline (eg, greater loneliness and social isolation, greater discrimination in online settings, and reported overzealous romantic ideation or limerence). Taken together, these findings suggest the need to acknowledge and address the role that psychological factors may play in YMSM’s DHI engagement. Given the correlation between psychological factors and HIV risk behaviors [39,40], researchers should explore how to address these psychological factors as part of the DHI implementation strategy to reduce the presence or severity of these HIV risk correlates while also creating opportunities to address other HIV risk factors in YMSM’s lives. For example, participants who self-report social isolation or online discrimination at baseline may benefit from access or nudges to intervention components focused on social support earlier on in the intervention, whereas those reporting limerence may benefit from intervention content and activities related to affect regulation earlier in the intervention.

The participants who viewed a greater number of sessions showed significant decreases in experienced discrimination in an online setting and internalized homophobia over time. Given the complexity of cognitive decision-making in health behavior [41], we do not know whether participants who had negative experiences in an online setting engaged with the intervention more than others to enhance their resilience, which could increase their ability to bounce back from those negative experiences and resolve internalized homophobia. For instance, it is plausible that participants who experienced discrimination in an online setting and had high levels of internalized homophobia viewed more sessions in an effort to enhance their resilience [42]. To examine whether these changes would improve DHI engagement, we encourage researchers to leverage innovations in research designs in future efforts. For instance, to detangle these complex behavior-change processes during a DHI, researchers may need to monitor the participants’ engagement and changes in their psychosocial behaviors in real time to understand these complex processes and respond by providing adequate intervention strategies. Just-in-time adaptive intervention designs [43] may facilitate these efforts given their ability to automatically detect changes in participants’ behaviors in real time and to deliver intervention components most relevant to the participants’ ongoing needs [36,44]. Just-in-time adaptive interventions have been used for various health behaviors, including addiction, mental health, and healthy diet [45]. Future intervention research examining whether optimized designs can increase DHI engagement is warranted.

The examination of various *paradata* metrics facilitates the understanding of accurate and meaningful engagement and outcome in DHIs. In this study, the amount of engagement (ie, sessions viewed) was significantly associated with internet use patterns, psychological facilitators and barriers, and partner-seeking correlates. However, the frequency of engagement (ie, the number of log-ins) was not associated with any of these factors. There is a tendency to assume the number of log-ins as the only *paradata* metric, but the results of this study highlight that the amount of intervention content participants consumed is a more meaningful measure to capture their behavior change. While traditional face-to-face interventions can control participants’ engagement through an intervention facilitator, DHIs offer no similar function to guarantee full use after the participants log in. However, we cannot conclude that the quality of engagement is better than the quantity of engagement. It is possible that meaningful correlates with the number of log-ins were not examined in this study, and meaningful *paradata* metrics could vary by study. Therefore, a rigorous measurement of *paradata* metrics to describe meaningful engagement in DHIs is needed. Future research investigating an array of *paradata* metrics to explain true engagement is warranted.

### Limitations

There are several limitations in this study. First, we selected 2 standardized metrics of engagement to understand frequency and amount as engagement domains, yet we recognize that other domains (eg, depth and duration) and metrics (eg, time spent in each component and use over time) may also be important to examine [9]. It may be worthwhile to consider how the proportion of engagement was linked to active learning (eg, interactive activities) compared to passive learning (eg, reading content) in future research. Unfortunately, we did not collect depth of engagement in our study. Future intervention studies examining how different engagement domains (in-depth engagement) may be related to DHI engagement are warranted. Second, we did not have a priori threshold to define optimal engagement for the number of sessions viewed and log-ins. In the absence of thresholds that may be used across studies, we will use the engagement data collected during this pilot trial to inform thresholds for a subsequent, large-scale clinical trial of the myDEx intervention. It also remains unclear whether comparable rates of engagement would be observed outside of a clinical trial. Therefore, future research examining how participants engage in myDEx, both within and outside of a clinical setting, is needed to characterize its potential as an intervention that may be used beyond a 3-month period. Third, we could not establish causal relationships between engagement and changes in characteristics. This study hypothesized that increased engagement led to changes in psychosocial and behavioral characteristics, but this can be interpreted in the opposite direction, such that changes in behavior lead to more engagement. Future research examining how changes in participants’ DHI engagement over time are related to the changes in hypothesized intervention mechanisms and key outcomes is warranted.

### Conclusions

*Paradata* analyses are a vital component of DHI evaluation. Determining intervention efficacy has proven challenging due to the absence of a consensus on what constitutes effective or meaningful engagement [16]. This study highlighted internet use patterns, psychological facilitators and barriers, and partner-seeking correlates associated with intervention engagement. Therefore, DHIs with personalized intervention components that consider the individuals’ differences could increase the overall engagement and efficacy of the intervention. Moreover, research identifying which components are popular in an intervention, which components work best for whom, and which intervention duration would derive the optimum result is warranted to increase the participants’ engagement.

## Appendix

Multimedia Appendix 1 Comparing descriptive statistics at baseline and 90-day follow-up.

Multimedia Appendix 2 Associations between baseline characteristics and myDEx engagement.

Multimedia Appendix 3 Changes in the participants’ behaviors based on myDEx engagement.

Multimedia Appendix 4 CONSORT-eHEALTH Checklist (V 1.6.1).

## Abbreviations

CAI: condomless anal intercourse
DHI: digital health intervention
HMP: healthMpowerment.org
MSM: men who have sex with men
YMSM: young men who have sex with men
