# Supplementary material for: Correlates of Engagement Within an Online HIV Prevention Intervention for Single Young Men Who Have Sex With Men: Randomized Controlled Trial
Source: JMIR Public Health Surveill. 2022 Jun 27;8(6):e33867. doi: 10.2196/33867 (PMC9274398; doi:10.2196/33867)
Supplement: Multimedia Appendix 1 [file publichealth_v8i6e33867_app1.docx]

**Multimedia Appendix 1.** Comparing descriptive statistics at baseline and 90-day follow-up.

| Characteristics | | Baseline  (N=120) | At 90-day Follow-up  (N=95) | P value^a^ |
| --- | --- | --- | --- | --- |
| **Demographic Characteristics** | |  |  |  |
| Age, mean (SD) | | 21.57 (1.81) | - |  |
| **Race, n(%)** | |  | - |  |
|  | White | 89 (74.17%) |  |  |
|  | Black | 18 (15.00%) |  |  |
|  | Native | 2 (1.67%) |  |  |
|  | Asian | 10 (8.33%) |  |  |
|  | Middle Eastern | 2 (1.67%) |  |  |
|  | Pacific | 0 (0%) |  |  |
|  | Other | 12 (10.0%) |  |  |
| Ethnicity - Latino, n(%) | | 35 (29.2%) | - |  |
| **Education, n(%)** | |  | - |  |
|  | Some high school | 4 (3.33%) |  |  |
|  | Graduated high school | 14 (11.67%) |  |  |
|  | Technical school | 4 (3.33%) |  |  |
|  | Associate degree | 2 (1.67%) |  |  |
|  | Some college | 50 (41.67%) |  |  |
|  | College | 34 (28.33%) |  |  |
|  | Some graduate school | 9 (7.50%) |  |  |
|  | Graduate school | 3 (2.50%) |  |  |
| **Internet Using Patterns** | |  |  |  |
| **Frequency of online dating to find a date, n(%)** | |  |  | .004 |
|  | Never | 15 (12.50%) | 32 (33.68%) |  |
|  | Once a month or less | 18 (15.00%) | 20 (21.05%) |  |
|  | 2-3 times a month | 28 (23.33%) | 18 (18.95%) |  |
|  | About once a week | 13 (10.83%) | 5 (5.26%) |  |
|  | 2-6 times a week | 20 (16.67%) | 12 (12.63%) |  |
|  | About once a day | 26 (21.67%) | 8 (8.42) |  |
| **Usefulness of online dating to find a date, n(%)** | |  |  | .193 |
|  | Not at all | 38 (31.67%) | 20 (31.75%) |  |
|  | Somewhat | 46 (38.33%) | 24 (37.10%) |  |
|  | Moderately | 26 (21.67%) | 14 (22.22%) |  |
|  | Very much | 10 (8.33%) | 5 (7.94%) |  |
| **Frequency of online dating to find a hook up, n(%)** | |  |  | .007 |
|  | Never | 24 (20.00%) | 44 (46.32%) |  |
|  | Once a month or less | 38 (31.67%) | 23 (24.21) |  |
|  | 2-3 times a month | 38 (31.67%) | 11 (11.58%) |  |
|  | About once a week | 11 (9.17%) | 8 (8.42%) |  |
|  | 2-6 times a week | 6 (500%) | 5 (5.26%) |  |
|  | About once a day | 3 (2.50%) | 4 (4.21%) |  |
| **Usefulness of online dating to find a hook up, n(%)** | |  |  | .065 |
|  | Not at all | 32 (26.67%) | 8 (15.69%) |  |
|  | Somewhat | 33 (27.50%) | 24 (47.06%) |  |
|  | Moderately | 30 (25.00%) | 9 (17.65%) |  |
|  | Very much | 25 (20.83%) | 10 (19.61%) |  |
| Discrimination when seeking partners online, mean (SD) | | 17.00 (9.70) | 3.25 (4.44) | <.001 |
| **Psychological Facilitators & Barriers** | |  |  |  |
|  | Internalized homophobia, mean (SD) | 7.65 (4.42) | 6.97 (4.48) | .333 |
|  | Loneliness, mean (SD) | 5.78 (1.82) | 5.55 (1.97) | .352 |
|  | Mental Health, mean (SD) | 8.94 (5.78) | 8.45 (5.86) | .901 |
|  | Self-Esteem, mean (SD) | 19.48 (5.75) | 20.49 (5.69) | .243 |
| **Partner-seeking correlates** | |  |  |  |
|  | Intimate romantic relationship, mean (SD) | 3.82 (0.33) | 3.82 (0.41) | .673 |
|  | Passionate romantic relationship, mean (SD) | 3.59 (0.40) | 3.62 (0.46) | .406 |
|  | Committed romantic relationship, mean (SD) | 3.72 (0.39) | 3.72 (0.45) | .823 |
|  | Limerence, mean (SD) | 22.93 (6.56) | 22.55 (7.56) | .421 |
| **Sexual risk behaviors** | |  |  |  |
|  | Decision balance to condom use, mean (SD) | -0.42 (0.96) | -0.26 (0.86) | .028 |
|  | Self-efficacy to use condom with a date, mean (SD) | 15.41 (4.38) | 15.08 (4.38) | .284 |
|  | Self-efficacy to use condom with hook up, mean (SD) | 16.82 (4.25) | 15.91 (4.25) | 0.059 |
|  | Number of sex partners, mean (SD) | 2.39 (2.73) | 1.15 (1.39) | <.001 |
|  | Receptive anal intercourse, mean (SD) | 1.03 (1.66) | 0.80 (1.00) | .152 |
|  | Insertive anal intercourse, mean (SD) | 0.86 (1.36) | 0.85 (1.05) | .693 |

^a^McNemar’s tests for categorical variables and paired t-tests for continuous variables.
