# Supplementary material for: Correlates of Engagement Within an Online HIV Prevention Intervention for Single Young Men Who Have Sex With Men: Randomized Controlled Trial
Source: JMIR Public Health Surveill. 2022 Jun 27;8(6):e33867. doi: 10.2196/33867 (PMC9274398; doi:10.2196/33867)
Supplement: Multimedia Appendix 2 [file publichealth_v8i6e33867_app2.docx]

**Multimedia Appendix 2.** Associations between baseline characteristics and myDEx engagement.

| Baseline Characteristics | | The number of logins | | | | The number of sessions viewed | | | |
| --- | --- | --- | --- | --- | --- | --- | --- | --- | --- |
|  |  | Univariate | | Multivariate | | Univariate | | Multivariate | |
|  |  | beta (SE) | *P* | beta (SE) | *P* | beta (SE) | *P* | beta (SE) | *P* |
| **Demographic Characteristics** | |  |  |  |  |  |  |  |  |
|  | Age | -0.001 (0.02) | .97 | - | - | -0.02 (0.02) | .34 | - | - |
|  | Race (Non-Hispanic White vs. other) | 0.02 (0.08) | .78 | - | - | 0.03 (0.07) | .70 | - | - |
|  | Ethnicity (Hispanic vs. other) | -0.16 (0.09) | .08 | - | - | -0.25 (0.08) | .002 | -0.27 (0.09) | <.001 |
|  | Education (some associate degree and above vs. some high, GED, or technical school) | 0.23 (0.11) | .04 | 0.22 (0.11) | .04 | 0.24 (0.10) | .01 | 0.22 (0.10) | .03 |
| **Internet Using Patterns** | |  |  |  |  |  |  |  |  |
|  | Frequency of online dating to find a date | 0.03 (0.02) | .21 | - | - | 0.03 (0.02) | .12 | - | - |
|  | Usefulness of online dating to find a date | 0.004 (0.04) | .92 | - | - | -0.06 (0.04) | .09 | - | - |
|  | Frequency of online dating to find a hook up | 0.07 (0.03) | .03 | 0.007 (0.05) | .89 | 0.06 (0.03) | .02 | -0.01 (0.05) | .80 |
|  | Usefulness of online dating to find a hook up | 0.09 (0.04) | .01 | 0.07 (0.05) | .13 | 0.14 (0.03) | <.001 | 0.13 (0.04) | .002 |
|  | Experienced discrimination in an online setting | -0.01 (0.004) | .01 | - | - | -0.01 (0.003) | .02 | -0.01 (0.004) | .01 |
| **Psychological Facilitators & Barriers** | |  |  |  |  |  |  |  |  |
|  | Internalized homophobia | -0.003 (0.01) | .97 |  |  | 0.002 (0.01) | .77 | - | - |
|  | Loneliness | 0.05 (0.02) | .02 | 0.04 (0.02) | .07 | 0.05 (0.02) | .01 | 0.06 (0.02) | .004 |
|  | Mental Health | 0.01 (0.01) | .32 | - | - | 0.01 (0.01) | .10 | - | - |
|  | Self-Esteem | 0.001 (0.01) | .87 | - | - | 0.01 (0.01) | .34 | - | - |
| **Partner-seeking correlates** | |  |  |  |  |  |  |  |  |
|  | Intimate romantic relationship | 0.08 (0.12) | .53 | - | - | 0.05 (0.11) | .67 | - | - |
|  | Passionate romantic relationship | 0.04 (0.10) | .72 | - | - | -0.04 (0.09) | .65 | - | - |
|  | Committed romantic relationship | 0.12 (0.11) | .26 | - | - | 0.15 (0.10) | .13 | - | - |
|  | Limerence | -0.01 (0.01) | .25 | - | - | -0.01 (0.01) | .02 | -0.02 (0.01) | .005 |
| **Sexual risk behaviors** | |  |  |  |  |  |  |  |  |
|  | Decision balance to condom use | 0.04 (0.04) | .38 | - | - | 0.07 (0.04) | .06 | - | - |
|  | Self-efficacy to use condom with a date | -0.004 (0.01) | .69 | - | - | 0.001 (0.01) | .89 | - | - |
|  | Self-efficacy to use condom with a hook up | -0.01 (0.01) | .54 | - | - | 0.004 (0.01) | .64 | - | - |
|  | Number of sex partners | 0.04 (0.01) | .01 | 0.02 (0.02) | .21 | 0.04 (0.01) | .001 | 0.01 (0.02) | .57 |
|  | Receptive anal intercourse | -0.01 (0.02) | .67 | - | - | -0.004 (0.02) | .87 | - | - |
|  | Insertive anal intercourse | 0.05 (0.03) | .07 | - | - | -0.04 (0.03) | .17 | - | - |
