# Supplementary material for: Correlates of Engagement Within an Online HIV Prevention Intervention for Single Young Men Who Have Sex With Men: Randomized Controlled Trial
Source: JMIR Public Health Surveill. 2022 Jun 27;8(6):e33867. doi: 10.2196/33867 (PMC9274398; doi:10.2196/33867)
Supplement: Multimedia Appendix 3 [file publichealth_v8i6e33867_app3.docx]

**Multimedia Appendix 3.** Changes in participants’ behaviors based on myDEx engagement.

| Changes in characteristics between baseline and at 90-day follow-up | | The number of logins^a^ | | The number of sessions view | | | |
| --- | --- | --- | --- | --- | --- | --- | --- |
|  |  | Univariate | | Univariate | | Multivariate | |
|  |  | beta (SE) | *P* value | beta (SE) | *P* value | beta (SE) | *P* value |
| **Changes in Internet Using Patterns** | |  |  |  |  |  |  |
|  | Frequency of online dating to find a date | 0.02 (0.02) | .32 | 0.02 (0.02) | .28 |  |  |
|  | Usefulness of online dating to find a date | 0.002 (0.04) | .96 | 0.06 (0.04) | .10 |  |  |
|  | Frequency of online dating to find a hook up | -0.01 (0.03) | .72 | 0.004 (0.02) | .87 |  |  |
|  | Usefulness of online dating to find a hook up | -0.07 (0.05) | .10 | -0.21 (0.04) | <.001 | -0.20 (0.05) | <.001 |
|  | Experienced discrimination in an online setting | 0.01 (0.04) | .13 | 0.002 (0.004) | .60 |  |  |
| **Changes in Psychological Facilitators & Barriers** | |  |  |  |  |  |  |
|  | Internalized homophobia | -0.02 (0.01) | .11 | -0.03 (0.01) | .01 | -0.06 (0.02) | <.001 |
|  | Loneliness | -0.002 (0.02) | .94 | -0.02 (0.02) | .45 |  |  |
|  | Mental Health | 0.01 (0.01) | .24 | -0.01 (0.01) | .19 |  |  |
|  | Self-Esteem | -0.01 (0.01) | .42 | 0.001 (0.01) | .89 |  |  |
| **Changes in Partner-seeking correlates** | |  |  |  |  |  |  |
|  | Intimate romantic relationship | 0.09 (0.16) | .57 | 0.29 (0.14) | .04 | 0.50 (0.30) | .10 |
|  | Passionate romantic relationship | -0.01 (0.10) | .88 | 0.05 (0.08) | .54 |  |  |
|  | Committed romantic relationship | 0.01 (0.09) | .96 | 0.08 (0.08) | .31 |  |  |
|  | Limerence | -0.01 (0.01) | .27 | 0.002 (0.01) | .74 |  |  |
| **Changes in Sexual risk behaviors** | |  |  |  |  |  |  |
|  | Decision balance to condom use | 0.08 (0.06) | .21 | 0.01 (0.05) | .86 |  |  |
|  | Self-efficacy to use condom with a date | 0.004 (0.01) | .65 | -0.01 (0.01) | .24 |  |  |
|  | Self-efficacy to use condom with a hook up | 0.03 (0.01) | .10 | -0.004 (0.01) | .63 |  |  |
|  | Number of sex partners | -0.01 (0.02) | .66 | 0.001 (0.01) | .93 |  |  |
|  | Receptive anal intercourse | 0.03 (0.03) | .40 | 0.02 (0.03) | .52 |  |  |
|  | Insertive anal intercourse | 0.01 (0.04) | .74 | 0.08 (0.04) | .02 | 0.07 (0.04) | .06 |

^a^Multivariate model was not examined due to non-significant associations in univariate analyses.
